# Multi-complexity measures of heart rate variability and the effect of vasopressor titration: a prospective cohort study of patients with septic shock

**DOI:** 10.1186/s12879-016-1896-1

**Published:** 2016-10-10

**Authors:** Samuel M. Brown, Jeffrey Sorensen, Michael J. Lanspa, Matthew T. Rondina, Colin K. Grissom, Sajid Shahul, V. J. Mathews

**Affiliations:** 1 Pulmonary and Critical Care, Intermountain Medical Center, Murray, UT USA; 2 Pulmonary and Critical Care, University of Utah School of Medicine, Salt Lake City, UT USA; 3 Internal Medicine, University of Utah Medical Center and School of Medicine and George E. Wahlen VA Medical Center, Salt Lake City, UT USA; 4 Molecular Medicine Program in the Eccles Institute of Human Genetics, Salt Lake City, UT USA; 5 Anesthesia and Critical Care, University of Chicago, Chicago, IL USA; 6 School of Electrical Engineering & Computer Science, Oregon State University, Corvallis, OR USA

**Keywords:** Sepsis, Shock, Physiological Variability, Heart Rate Variability

## Abstract

**Background:**

Septic shock is a common and often devastating syndrome marked by severe cardiovascular dysfunction commonly managed with vasopressors. Whether markers of heart rate complexity before vasopressor up-titration could be used to predict success of the up-titration is not known.

**Methods:**

We studied patients with septic shock requiring vasopressor, newly admitted to the intensive care unit. We measured the complexity of heart rate variability (using the ratio of fractal exponents from detrended fluctuation analysis) in the 5 min before all vasopressor up-titrations in the first 24 h of an intensive care unit (ICU) admission. A successful up-titration was defined as one that did not require further up-titration (or decrease in mean arterial pressure) for 60 min.

**Results:**

We studied 95 patients with septic shock, with a median APACHE II of 27 (IQR: 20–37). The median number of up-titrations, normalized to 24 h, was 12.2 (IQR: 8–17) with a maximum of 49. Of the up-titrations, the median proportion of successful interventions was 0.28 (IQR: 0.12–0.42). The median of mean arterial pressure (MAP) at the time of a vasopressor up-titration was 66 mmHg; the average infusion rate of norepinephrine at the time of an up-titration was 0.11 mcg/kg/min. The ratio of fractal exponents was not associated with successful up-titration on univariate or multivariate regression. On exploratory secondary analyses, however, the long-term fractal exponent was associated (*p =* 0.003) with success of up-titration. Independent of heart rate variability, MAP was associated (*p <* 0.001) with success of vasopressor up-titration, while neither Sequential Organ Failure Assessment (SOFA) nor Acute Physiology and Chronic Health Evaluation II (APACHE II) score was associated with vasopressor titration.

**Conclusions:**

Only a third of vasopressor up-titrations were successful among patients with septic shock. MAP and the long-term fractal exponent were associated with success of up-titration. These two, complementary variables may be important to the development of rational vasopressor titration protocols.

**Electronic supplementary material:**

The online version of this article (doi:10.1186/s12879-016-1896-1) contains supplementary material, which is available to authorized users.

## Background

Septic shock, the life-threatening manifestation of severe infection, is associated with a near-term mortality of 20-30 % [1–3]. Current consensus emphasizes early intervention to control sepsis [4, 5], even if recent assumptions about how best to do so have changed in the aftermath of three large studies [6–8] that failed to demonstrate benefit to the prior paradigm of “early goal-directed therapy,” which had used multiple, integrated therapies to optimize oxygen delivery [4]. While early antibiotics and source control are nearly universally accepted, other details of early sepsis management are not well understood.

Cardiovascular failure is common in sepsis, often resulting in multiple organ dysfunction syndrome (MODS), which is frequently fatal. Vascular leakage causes a decrease in cardiac preload, and arterial dilation causes decrease in cardiac afterload. Microvascular dysfunction is common, and cardiac function is often impaired [9, 10]. Together these phenomena contribute to a state of hypoperfusion that propagates septic MODS. Despite the lack of clear physiological targets to track, current mainstays of treatment for septic shock are volume expansion and vasopressor administration, designed to increase cardiac preload (and thereby cardiac output) and to raise the systemic pressure for perfusion.

Vasopressors, primarily catecholamine hormones such as norepinephrine, are generally administered in shock to elevate the blood pressure (and recruit unstressed volume in capacitance veins to increase cardiac preload), in order to assure perfusion of coronary arteries as well as distal organs. While vasopressors are an important therapy in patients with shock, it is unclear how best to titrate them. While something like consensus supports targeting a mean arterial pressure between 60–75 mmHg, most clinicians adjust infusion rates by hand in hopes of achieving whatever target is locally preferred [11].

Little work has been done to identify best methods of vasopressor titration. Small studies have explored predictors of the safety of vasopressor weaning in patients with septic shock [12, 13] One possibility is that the degree of dysfunction of the baroreflex system might be informative. An evolutionarily ancient system that maintains cardiovascular homeostasis, the baroreflex is central to the response to septic MODS. The baroreflex adjusts vascular elastance, cardiac contractility, and heart rate. The interval between heartbeats is determined by the sinus node, which reflects the summary effects of multiple inputs into the baroreflex system. Analysis of the changes in instantaneous heart rate over time, termed “heart rate variability (HRV),” provides an important window into autonomic control of the baroreflex system in primary cardiac disease [14–16], acute trauma [17] and sepsis [18, 19]. Notably, in health HRV exhibits nonlinear patterns of complexity, including characteristics of chaotic systems. One key aspect of such chaotic systems is that patterns are conserved across levels of scale, what is called fractal self-similarity across time scales [20, 21]. Heart rate variability exhibits complex patterns that may give insight into the interdependence of the autonomic nervous system and inflammation in sepsis [22]. Complexity analyses have been identified as a key research priority in critical illness [23, 24]. Our prior work demonstrated that loss of complexity in heart rate variability is associated with worse outcomes in severe sepsis and septic shock [25]. We therefore hypothesized that the complexity of heart rate variability in the 5 min before an increase in vasopressor infusion rates would be associated with the success of that vasopressor increase.

## Methods

### Setting

We studied patients admitted to either of two ICUs, the 24-bed Shock Trauma ICU, and the 12-bed Respiratory ICU, at Intermountain Medical Center, a 450-bed tertiary-care, academic hospital in Murray, Utah, USA. Typical clinical practice in the study ICUs is to titrate vasopressors for a mean arterial pressure of 60-65 mmHg.

### Patients

We prospectively identified adult (>15 years of age) patients with septic shock (as defined in consensus guidelines [26]) admitted to study ICUs from June 2012 to March 2015. We excluded pregnant patients, patients with do-not-resuscitate/do-not-intubate (DNR/DNI) orders at the time of ICU admission, and patients with non-sinus rhythm. We only included patients the first time they were admitted to a study ICU with sepsis during the study period. Patients were only included at the time of their initial admission to the ICU; we excluded patients who developed sepsis after their admission to the ICU.

We restricted the study to those patients who had at least one increased infusion rate of vasopressors in the first 24 h of their ICU admission for septic shock.

### Clinical data

We calculated admission APACHE II [27] and SOFA [28] scores in all study patients. Infusion rates of vasopressors (norepinephrine, epinephrine, dopamine, phenylephrine, and vasopressin) are automatically uploaded in real-time to the hospital electronic medical record (EMR) as part of routine clinical care. We analyzed all vasopressors administered during the first six hours after ICU admission, converting them to norepinephrine equivalent dosages according to standard equivalencies [29]. The EMR also stores the values of heart rate and blood pressure at 30-s intervals. These values represent 8-beat moving medians, obtained from the Philips bedside monitors.

### Physiological data acquisition and processing

**Fig. 1:**
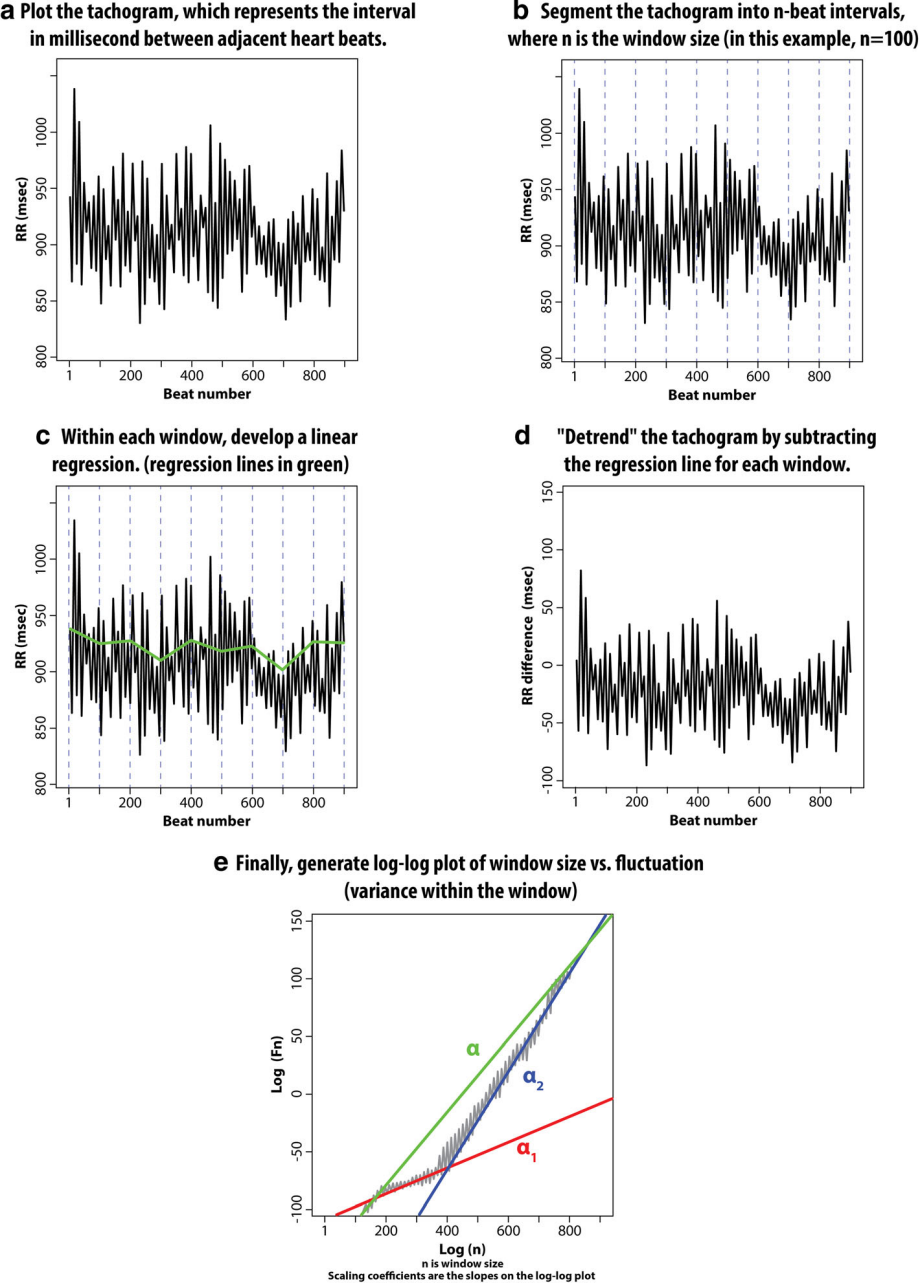
Depiction of detrended fluctuation analysis

Data were downloaded from Philips Intellivue Monitors via the Research Data Export (RDE) functionality. RDE provides 125-Hz digitized tracings of continuous electrocardiographic monitoring as well as identification of fiducial points (the moment when the QRS complex occurs). Using those fiducial points, we processed the data within Continuous Individual Multiorgan Variability Analysis (CIMVA) [30]. We used 5 min analytical windows for CIMVA, incremented 2.5 min at each step. For each vasopressor uptitration we used the complexity measures from the 5 min before the vasopressor uptitration occurred. Our pre-specified primary predictors were entropy and the ratio of exponents from detrended fluctuation analysis, based on prior work [25, 31]. (We depict an example detrended fluctuation analysis in Figure 1.) Secondarily, we considered all CIMVA complexity measures as potential candidates. The CIMVA complexity measures represent the range of validated complexity measures that have been or are likely to be relevant to human physiological monitoring. These measures, or combinations thereof, have been evaluated in a variety of acute illness states [30–32]. CIMVA measures were not transformed, combined, or otherwise modified in this study. We considered secondary analyses to be exploratory and hypothesis generating.

We only considered one vasopressor up-titration in any five-minute period: if a vasopressor up-titration occurred within 5 min after another, the latter up-titration was used. Only up-titrations ≥ 0.01 mcg/kg/min were considered. We excluded from consideration any up-titration followed by a down-titration within 1.5 min.

### Clinical outcomes

We defined, *a priori*, successful vasopressor up-titration as maintenance of mean arterial pressure > 60 mm Hg for 60 min after the increased dosage without further titration during those 60 min.

### Statistical methods

Central tendencies and variance were expressed as mean (standard deviation) or median (inter-quartile range) as indicated by normality of the data. Statistical analysis and hypothesis testing were performed within the R statistical package version 3.1.3 [33], with OpenBUGS 3.2.3 used for Bayesian analyses. Generalized linear mixed models (GLMM) were used to account for the dependence structure within each patient, as multiple up-titrations could occur per patient. A distinctive feature of repeated measures is that they are clustered within individuals. Observations within a cluster typically exhibit positive correlation, the presence of which violates the crucial assumption of independence in standard statistical models. The degree of clustering can be measured, and appropriate statistical models for clustered data must account for the degree of clustering/non-independence [34]. Generalized linear mixed models (GLMM) can account for lack of independence within clustered data by assigning each cluster of repeated measures its own intercept, the whole of which are constrained to be normally distributed. In this way, the proportion of correlation due to clustering can be measured, allowing identification of reliable parameter estimates [35]. We implemented univariate, multilevel logistic regression with random intercepts, predicting the probability of a vasopressor up-titration being successful. Complexity measures were centered and scaled to facilitate convergence of regression models. Using bootstrapping to estimate power, we calculated that with a sample size of 95, we had 80 % power with two-tailed alpha = 0.05 to detect a 0.27 absolute difference in the ratio of detrended fluctuation analysis (DFA) exponents between successful and unsuccessful vasopressor up-titrations.

## Results

**Figure 2:**
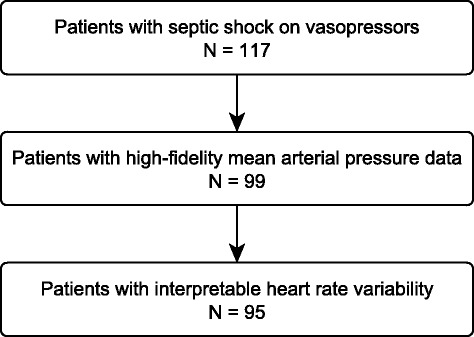
Selection of patients for analysis

Of 117 patients with septic shock requiring vasopressor infusions, 95 (81 %) had adequate arterial blood pressure data and interpretable HRV complexity data. Figure 2 depicts the flow of patients through screening and analysis. Table 1 depicts patient demographics, measures of disease severity, and sources of sepsis. Patients had a median APACHE II score of 27 (20–37). Patients received vasopressors for median 23 h (20–24), with four individuals receiving vasopressors for 500 h or more.

**Table 1:** Descriptive statistics

	Median	(IQI)
Age (years)	59.0	(47.5 to 66.5)
SOFA	11	(8 to 13)
APACHE II	27	(20 to 36.5)
Mean heart rate (bpm)	103	(93 to 119)
MAP at up-titration (mmHg)	66	(61 to 72)
NEE infusion rate prior to intervention (*μ*g/kg/min)	0.11	(0.02 to 0.28)
Number of interventions (normalized to 24 h)	12.2	(8.0 to 17.2)
Number of successful interventions (normalized to 24 h)	3.0	(1.3 to 4.8)
Ratio of successful interventions (normalized to 24 h)	0.28	(0.12 to 0.42)
Duration of NEE administration (hours)	22.8	(19.9 to 23.6)
	Proportion	(n)
Female	52.6 %	(50)
MAP *≤* 65 at interventions (mmHg)	49.0 %	(472)
MAP *≤* 60 at interventions (mmHg)	23.0 %	(222)
Source of sepsis		
Pneumonia	32.6 %	(31)
Abdominal	29.5 %	(28)
Soft tissue	16.8 %	(16)
Urinary	10.5 %	(10)
Uncertain	3.2 %	(3)
Bacteremia	2.1 %	(2)
Joint	2.1 %	(2)
Endocarditis	1.1 %	(1)
Line infection	1.1 %	(1)
Other	1.1 %	(1)

A total of 964 up-titrations among the 95 patients were analyzed. The median number of up-titrations per patient, normalized to 24 h, was 12.2 (IQR: 8–17) with a maximum of 49. Of the up-titrations, the median proportion of successful interventions was 0.28 (IQR: 0.12–0.42). In four patients with at least two up-titrations, 100 % of up-titrations were successful. The median of the mean arterial pressure (MAP) at the time of a vasopressor up-titration was 66 mmHg; the average infusion rate of norepinephrine at the time of an up-titration was 0.11 mcg/kg/min.

### Primary analysis

For our pre-specified primary analysis, we evaluated the ratio of fractal scaling coefficients from detrended fluctuation analysis and Sample Entropy. Neither was associated with successful up-titration on univariate analysis or on bivariate analysis controlling for MAP at the time of up-titration. When controlling for age, sex, and admission APACHE II, there was also no significant association. The results for the model evaluating the ratio of scaling coefficients is displayed in Table 2, while the results for the model including sample entropy are displayed in Table 3.

**Table 2:** Results of primary regression analyses for fractal exponents

Variable	OR	95 % CI	*p*
Ratio of exponents	1.055	0.890 –1.25	0.54
MAP	1.3	1.09 –1.56	0.004
Delta NEE	1.3	0.9 –1.89	0.163
Age	0.96	0.75 –1.23	0.741
Male	0.77	0.46 –1.29	0.323
SOFA	1.04	0.93 –1.17	0.476
APACHE II	0.97	0.94 –1.01	0.133

**Table 3:** Results of primary regression analysis for sample entropy

Variable	OR	95 % CI	*p*
Sample entropy	0.97	0.81 –1.16	0.76
MAP	1.3	1.08 –1.55	0.005
Delta NEE	1.29	0.89 –1.86	0.177
Age	0.95	0.74 –1.22	0.708
Male	0.78	0.46 –1.31	0.341
SOFA	1.04	0.93 –1.17	0.468
APACHE II	0.97	0.93 –1.01	0.117

### Exploratory secondary analyses

In prespecified exploratory analyses, we evaluated the association of all CIMVA complexity measures against the primary outcome. Notably, the overall fractal exponent (*p =* 0.003) and the long-term fractal exponent (*p =* 0.003) were significantly associated with success of vasopressor titration, effects which persisted after control for APACHE II, MAP, age and sex (*p =* 0.007 for both). We display these findings in Table 4. Additional file 1: Table S1, in the online data supplement, displays the candidate predictors with odds ratios and p values for univariate and multivariate associations.

**Table 4:** Results of exploratory regression analyses for additional results from detrended fluctuation analysis

	Univariate		Bivariate^a^		Multivariate^b^	
Variable	OR (95 % CI)	*p* value	OR (95 % CI)	*p* value	OR (95 % CI)	*p* value
Long-term fractal exponent	1.3 (1.1–1.6)	0.003	1.3 (1.1–1.6)	0.005	1.3 (1.1–1.6)	0.007
Overall fractal exponent	1.4 (1.1–1.7)	0.003	1.3 (1.1–1.6)	0.005	1.3 (1.1–1.6)	0.007

^a^The bivariate model controlled for mean arterial pressure (MAP)

^b^The multivariate model controlled for MAP, magnitude of vasopressor up-titration, APACHE II, SOFA, age, and sex

The distributions of the long-term fractal exponent differed between up-titrations that were successful (*n =* 229, median 1.11, IQR 0.926 to 1.32) and those that were unsuccessful (*n =* 735, median 1.05, IQR 0.739 to 1.25) (*p <* 0.001). The distributions of the overall fractal exponent also differed between up-titrations that were successful (median 1.02, IQR 0.800 to 1.28) and those that were unsuccessful (median 0.956, IQR 0.597 to 1.20) (Wilcoxon rank sum test *p <* 0.001).

In an exploratory analysis of other clinical predictors of successful vasopressor up-titration, MAP before the up-titration was strongly associated (*p <* 0.001) with success of the up-titration; trends toward association between the size of the up-titration (*p =* 0.09) and APACHE II score (*p =* 0.08) and success of up-titration were also observed. None of age, sex, and SOFA score was associated with success of vasopressor up-titration.

## Discussion

In this prospective study of patients admitted to the ICU with septic shock, an array of heart rate complexity measures were not significantly associated with the success of a vasopressor up-titration after adjustment for multiple comparisons. While we had hypothesized, on the basis of prior work on early sepsis resuscitation [25], that the ratio of fractal scaling coefficients would be associated with outcome, the overall and long-term coefficients, rather than the ratio or the short-term coefficient, appeared promising on an exploratory analysis.

The long-term and overall fractal exponents were suggestive on exploratory analysis, even after control for severity of illness. A higher exponent was associated with a higher probability of success. This higher exponent represents an increase in variability (“fluctuation”) over time intervals longer than approximately one minute (i.e., around 100 heart beats), with greater variability over longer time intervals. It may be that patients in whom that variability is lower are less likely to respond to vasopressor up-titrations because they have more severe vasoplegia or, contrarily, have a defect in cardiac rather than vascular function. Recent work on the mismatch between ventricular and arterial elastance (“ventriculo-arterial decoupling”) in septic shock suggests that certain patients may differentially benefit from vasoconstriction versus fluid loading or inotropic support. [36] It is possible that in our cohort some vasopressor up-titrations were unsuccessful because of ventriculo-arterial decoupling. Future work should explore possible relationships between detrended fluctuation analysis and ventriculo-arterial decoupling. In our prior work, higher long-term fractal exponents at the time of ICU admission were associated with a lower probability of vasopressor independence at 24 h [25]. Our current observations also suggest that the temporal arc of septic shock may be relevant to the success of vasopressor up-titrations in the short term versus liberation from vasopressor therapy over the intermediate term. The precise nature of this association is worth exploring if future work validates these current observations.

Beyond evaluation of specific predictors, our study provides a useful snapshot of current vasopressor titration practices. On average, doses are adjusted approximately a dozen times in a 24-h period. While the nominal practice in the study ICUs is to target 65 mmHg, many adjustments are made at a moment when the MAP is slightly higher, perhaps representing a desire to never fall below the target MAP. Our study also documents the reality that only about a third of vasopressor up-titrations were successful. This raises the possibility that improved predictive models could facilitate higher-quality approaches to the titration of vasopressors. On the basis of our data, the MAP at the time of up-titration should be incorporated into such predictive models. It may be that the MAP is the most important predictor, and heart rate variability metrics may contribute relatively little. In many if not most control systems, the distance between the current and desired level of a parameter of interest is a key determinant of the required input. Future work should address whether the distance between desired and current blood pressure could guide vasopressor titration.

Early work in dogs has suggested the possibility that automated control of vasopressor infusions may be possible [37]. To our knowledge, this work has not been replicated in humans. Merouani and colleagues successfully used fuzzy logic for a vasopressor weaning protocol. [13] In uncontrolled work, the dynamic arterial elastance (a measure of vasodilation) of patients in septic shock predicted that the patients were ready for weaning from norepinephrine [12].

With ongoing interest in understanding optimal MAP goals [38], our results suggest the importance of more explicit protocols given how often an up-titration is not effective and how often up-titrations occur when the MAP is higher than the target.

Prior work has suggested that complexity metrics may be useful for clinical management of patients with or at risk for infection. Among premature infants, a complexity-monitoring system focused on entropy measurements demonstrated improved outcomes, primarily as an early identification system for sepsis [31]. A composite complexity measure was associated with greater probability of extubation success in a multi-center study [32].

A strength of this study is our prespecified analytic approach, including primary predictors and clinical outcomes. Our use of prespecified primary analyses decreases the chance of Type 1 statistical error. Our non-definitive, exploratory analyses suggest that it would be reasonable to evaluate the overall and long-term fractal exponents in another cohort to ensure that the null hypothesis has not been accepted incorrectly.

Limitations of this study include the fact that in study ICUs vasopressor titration is predominantly performed by the bedside nurse, at her/his discretion. This practice likely results in unmeasured inter- and intra-clinician variation, which we were unable to control for.

## Conclusions

In summary, the long-term fractal exponent of heart rate variability and mean arterial pressure at the time of a vasopressor up-titration may be predictive of the success of that vasopressor up-titration. Further work is indicated to improve the rationality and predictability of vasopressor titration for patients with septic shock.

## Additional file

Additional file 1: Table S1. Online Data Supplement. Evaluated complexity measures and association with success of vasopressor titration on univariate and multivariate analyses. (DOCX 30 kb)

## Abbreviations

APACHE II: Acute Physiology and Chronic Health Evaluation II
CIMVA: Continuous Individual Multiorgan Variability Analysis
DFA: Detrended Fluctuation Analysis
DNR/DNI: Do-Not-Resuscitate/Do-Not-Intubate
EMR: Electronic Medical Record
GLMM: Generalized linear mixed models
HRV: Heart rate variability
ICU: Intensive care unit
IQR: InterQuartile Range
MAP: Mean arterial pressure
MODS: Multiple organ dysfunction syndrome
RDE: Research data export
SOFA: Sequential organ failure assessment
