# Supplementary material for: Multi-complexity measures of heart rate variability and the effect of vasopressor titration: a prospective cohort study of patients with septic shock
Source: BMC Infect Dis. 2016 Oct 10;16:551. doi: 10.1186/s12879-016-1896-1 (PMC5057204; doi:10.1186/s12879-016-1896-1)
Supplement: Additional file 1: Table S1. — Online Data Supplement. Evaluated complexity measures and association with success of vasopressor titration on univariate and multivariate analyses. (DOCX 30 kb) [file 12879_2016_1896_MOESM1_ESM.docx]

Multi-complexity measures of heart rate variability and the effect of vasopressor titration: a prospective cohort study of patients with septic shock

Samuel M. Brown, MD MS FASE^1,2^ (Samuel.Brown@imail.org), Jeff Sorensen^1^ (Jeff.Sorensen@imail.org), Michael J. Lanspa, MD^1,2^ (Michael.Lanspa@imail.org), Matthew T. Rondina, MD^3^ (Matthew.Rondina@hsc.utah.edu), Colin K. Grissom, MD^1,2^ (Colin.Grissom@imail.org), Sajid Shahul, MD^4^, V.J. Mathews, PhD^5^ (Mathews@ece.utah.edu)

^1^Pulmonary and Critical Care, Intermountain Medical Center, Murray, UT USA

^2^Pulmonary and Critical Care, University of Utah School of Medicine, Salt Lake City, UT USA

^3^Internal Medicine, University of Utah Medical Center and School of Medicine, Salt Lake City, UT USA

^4^Anesthesia and Critical Care, University of Chicago, Chicago, IL USA

^5^School of Electrical Engineering & Computer Science, Oregon State University, Corvallis, OR USA

**ONLINE DATA SUPPLEMENT**

| **eTable 1. Evaluated complexity measures and association with success of vasopressor titration on univariate and multivariate analyses** | | | | | |
| --- | --- | --- | --- | --- | --- |
| **Complexity Measure** | **Description** | **Univariable OR (95% CI)** | **Univariable  p-value** | **Multivariable OR (95% CI)** | **Multivariable p-value** |
| DFA.Alpha.2 | Computed as overall root-mean-square fluctuation F(n) of integrated and detrended event signals on multiple timescales n. Linear log10-log10 plot of F(n) versus n indicates fractal scaling. Fitted to range log10(n)>=1.2, corresponding to long-term fluctuations | 1.3 (1.1-1.6) | 0.003 | 1.3 (1.1 to 1.6) | 0.007 |
| DFA.Alpha | Same as above but fitted to entire range. | 1.4 (1.1-1.7) | 0.003 | 1.3 (1.1-1.6) | 0.007 |
| SymDp2_2 | Symbolic dynamics: percentage of 2 variation sequences, non-uniform case | 0.7 (0.5-0.9) | 0.005 | 0.7 (0.5-0.9) | 0.008 |
| Mobility | Hjorth Mobility: The mobility parameter represents the mean frequency, or the proportion of standard deviation of the power spectrum. | 0.7 (0.5-0.9) | 0.016 | 0.7 (0.5-0.9) | 0.019 |
| higu | Higuchi scaling exponent | 0.8 (0.7-1) | 0.028 | 0.8 (0.7-1) | 0.024 |
| SymDp2_1 | Symbolic dynamics: percentage of 2 variations sequences, uniform case | 0.8 (0.7-1) | 0.026 | 0.8 (0.7-1) | 0.031 |
| Complexity | Hjorth Complexity: The complexity parameter represents the change in frequency. The parameter compares the signal's smiliarity to a pure sine wave, where the value converges to 1 if the signal is more similar. | 1.2 (1-1.4) | 0.105 | 1.2 (1-1.5) | 0.043 |
| Power.Law.Goodness .of.Fi.LombScargle |  | 0.8 (0.7-1) | 0.042 | 0.8 (0.7-1) | 0.045 |
| Poincaré.SD1 | The Poincaré representation of an input event series is a scatter plot in which each point is plotted against its neighbour in a Cartesian plane. | 0.8 (0.6-1) | 0.045 | 0.8 (0.6-1) | 0.047 |
| RMSSD | Root-mean squared of successive differences. This measures the variability in successive event values. | 0.8 (0.6-1) | 0.045 | 0.8 (0.6-1) | 0.047 |
| sdDiff | Standard deviation of the differences | 0.8 (0.6-1) | 0.045 | 0.8 (0.6-1) | 0.047 |
| HF.Power.LombScargl e | HF is the area under the spectrum in the high frequency band 0.15-0.40Hz | 0.8 (0.7-1) | 0.064 | 0.8 (0.7-1) | 0.059 |
| LF.Power.LombScargle | LF is the area under the spectrum in the low frequency band 0.04-0.15Hz | 1.2 (1-1.4) | 0.043 | 1.2 (1-1.4) | 0.071 |
| formF | Form factor | 1.2 (1-1.4) | 0.07 | 1.2 (1-1.4) | 0.083 |
| SymDp0_2 | Symbolic dynamics: percentage of 0 variations sequences, non-uniform case | 1.2 (1-1.5) | 0.082 | 1.2 (1-1.5) | 0.084 |
| hldSWV | Scaled windowed variance | 1.2 (1-1.4) | 0.087 | 1.2 (1-1.4) | 0.093 |
| ARerr | Predictive feature: error from an Autoregressive model | 0.8 (0.7-1) | 0.093 | 0.8 (0.6-1) | 0.096 |
| CSI | Poincaré plot cardiac sympathetic index | 1.2 (1-1.4) | 0.083 | 1.2 (1-1.4) | 0.098 |
| LF.HF.ratio | Low frequency - high frequency ratio | 1.1 (1-1.4) | 0.126 | 1.2 (1-1.4) | 0.105 |
| hRDRA | Rescaled detrended range analysis | 1.2 (1-1.4) | 0.091 | 1.2 (1-1.4) | 0.119 |
| LeeP | Lee parameter | 0.9 (0.7-1.1) | 0.171 | 0.8 (0.7-1) | 0.119 |
| Coefficient.of. variation | The standard deviation of the events in the analysis window, normalized by the mean. (SD/mean) | 0.9 (0.7-1.1) | 0.183 | 0.8 (0.7-1.1) | 0.127 |
| VLF.Power.LombScar gle | Very low frequency power (0.003Hz-0.04Hz) | 1.2 (1-1.4) | 0.117 | 1.2 (1-1.4) | 0.131 |
| DFA.Alpha.1 | Computed as overall root-mean-square fluctuation F(n) of integrated and detrended event signals on multiple timescales n. Linear log10-log10 plot of F(n) versus n indicates fractal scaling. Fitted to range log10(n)<=1.2, corresponding to long-term fluctuations | 1.2 (1-1.4) | 0.114 | 1.2 (1-1.4) | 0.137 |
| PSeo | Plotkin and Swamy energy operator average energy | 1.2 (1-1.4) | 0.108 | 1.1 (1-1.4) | 0.138 |
| dlmean | Recurrence quantification analysis: mean diagonal line | 0.8 (0.6-1.1) | 0.201 | 0.8 (0.5-1.1) | 0.142 |
| Wavelet.AUC | Wavelet analysis is used to represent an input signal in time and frequency domains simultaneously. A savelet basis implemented as Daubechies least assymetric -8-tap filters are used in the maximal overlap wavelet decomposition. At each time scale, wavelet spectral density is computed and the area under this curve (AUC) is calculated. For 5 min HRV analysis, 5 scales is used. | 0.9 (0.7-1.1) | 0.261 | 0.9 (0.7-1.1) | 0.145 |
| gcount | Grid transformation feature: grid count | 0.9 (0.7-1.1) | 0.293 | 0.9 (0.7-1.1) | 0.157 |
| SymDp0_1 | Symbolic dynamics: percentage of 0 variations sequences, uniform case | 1.1 (0.9-1.3) | 0.177 | 1.1 (1-1.4) | 0.16 |
| CVI | Poincaré plot cardiac vagal index | 0.9 (0.7-1.1) | 0.267 | 0.9 (0.7-1.1) | 0.162 |
| tTime | Recurrence quantification analysis: trapping time | 0.9 (0.7-1.1) | 0.268 | 0.9 (0.7-1.1) | 0.196 |
| IoV | Index of variability distance from a Poisson distribution | 1.1 (0.9-1.3) | 0.279 | 1.1 (0.9-1.4) | 0.206 |
| SDLEalpha | Scale dependent Lyapunov exponent slope | 0.9 (0.8-1.1) | 0.192 | 0.9 (0.8-1.1) | 0.21 |
| KSe | Kolmogorov-Sinai entropy | 0.9 (0.7-1.1) | 0.239 | 0.9 (0.7-1.1) | 0.215 |
| DM2 | Dynamical moment of the second order | 0.8 (0.5-1.2) | 0.255 | 0.8 (0.5-1.2) | 0.232 |
| Power.Law.Goodness .of.Fit...Frequency.based |  | 0.9 (0.8-1.1) | 0.269 | 0.9 (0.8-1.1) | 0.242 |
| Mean.rate | 60/Mean | 0.9 (0.7-1.1) | 0.167 | 0.9 (0.7-1.1) | 0.254 |
| Mean | Average of the window | 1.2 (0.9-1.4) | 0.177 | 1.1 (0.9-1.4) | 0.259 |
| mDiff | Mean of the differences | 0.9 (0.8-1.1) | 0.226 | 0.9 (0.8-1.1) | 0.263 |
| vlmax | Recurrence quantification analysis: maximum vertical line | 0.9 (0.8-1.1) | 0.373 | 0.9 (0.7-1.1) | 0.283 |
| Activity | Hjorth Activity: The activity parameter represents the signal power, the variance of a time function. This can indicate the surface of power spectrum in the frequency domain. | 1.1 (0.9-1.4) | 0.205 | 1.1 (0.9-1.4) | 0.291 |
| Standard.Deviation | The standard deviation of the events in the analysis window | 0.9 (0.8-1.1) | 0.445 | 0.9 (0.7-1.1) | 0.305 |
| AsymI | Multiscale time irreversibility asymmetry index | 1.1 (0.9-1.3) | 0.338 | 1.1 (0.9-1.3) | 0.323 |
| DM3sb | Dynamical moment of the third order along the secondary bisector | 1.1 (0.9-1.3) | 0.282 | 1.1 (0.9-1.3) | 0.33 |
| SymDfw_1 | Symbolic dynamics: forbidden words, uniform case | 1.1 (0.9-1.3) | 0.434 | 1.1 (0.9-1.3) | 0.359 |
| pDpR | Recurrence quantification analysis: determinism/recurrences | 1.1 (0.9-1.3) | 0.359 | 1.1 (0.9-1.3) | 0.366 |
| SymDce_2 | Symbolic dynamics: modified conditional entropy, non-uniform case | 0.9 (0.7-1.1) | 0.329 | 0.9 (0.7-1.1) | 0.369 |
| Largest.Lyapunov. exponent | Lyapunov exponents estimate the degree of chaos present in the dynamical system described by the input event series (and represented in the state-space). The parameters m (embedding dimension) and t (time delay) are used to re-create the phase space from the time series, according to Takens' theorem. For HRV, m=5, t=3 | 0.9 (0.8-1.1) | 0.38 | 0.9 (0.8-1.1) | 0.374 |
| MultiFractal_c2 | Multifractal spectrum cumulant of the second order | 1.1 (0.9-1.3) | 0.358 | 1.1 (0.9-1.3) | 0.377 |
| DFA.AUC | Area under the DFA curve | 0.9 (0.8-1.2) | 0.587 | 0.9 (0.7-1.1) | 0.381 |
| DM3x | Dynamical moment of the third order along the x axes | 1.1 (0.9-1.3) | 0.331 | 1.1 (0.9-1.3) | 0.383 |
| DM3y | Dynamical moment of the third order along the y axes | 1.1 (0.9-1.3) | 0.332 | 1.1 (0.9-1.3) | 0.392 |
| Power.Law.Slope...Fre quency.based |  | 1 (0.8-1.1) | 0.576 | 0.9 (0.8-1.1) | 0.414 |
| shannEn | Shannon entropy | 1 (0.8-1.2) | 0.644 | 0.9 (0.7-1.1) | 0.425 |
| Power.Law.Goodness .of.Fit...Histogram.based |  | 1.1 (0.9-1.3) | 0.399 | 1.1 (0.9-1.3) | 0.455 |
| pR | Recurrence quantification analysis: percentage of recurrences | 0.9 (0.8-1.1) | 0.423 | 0.9 (0.8-1.1) | 0.455 |
| eScaleE | Embedding scaling exponent | 0.9 (0.8-1.1) | 0.382 | 0.9 (0.8-1.1) | 0.465 |
| Correlation.dimension | | 0.9 (0.7-1.1) | 0.389 | 0.9 (0.8-1.1) | 0.486 |
| HF.Power | High frequency power | 1.1 (0.9-1.3) | 0.403 | 1.1 (0.9-1.3) | 0.486 |
| DFA.Alpha.ratio | DFA Alpha 1/ DFA Alpha 2 | 1.1 (0.9-1.3) | 0.537 | 1.1 (0.9-1.3) | 0.488 |
| sgridAND |  | 1 (0.8-1.1) | 0.634 | 0.9 (0.8-1.1) | 0.493 |
| Power.Law.Y.Intercep t...Frequency.based |  | 1 (0.8-1.2) | 0.815 | 0.9 (0.8-1.1) | 0.497 |
| Power.Law.Slope...Hi stogram.based |  | 0.9 (0.8-1.1) | 0.418 | 0.9 (0.8-1.1) | 0.512 |
| Kurtosis | Kurtosis is a measure of whether the data are peaked or flat relative to a normal distribution. | 1 (0.9-1.2) | 0.601 | 1.1 (0.9-1.2) | 0.523 |
| IQR | Interquartile range | 0.9 (0.8-1.2) | 0.613 | 0.9 (0.7-1.2) | 0.523 |
| Power.Law.X.Interce pt.LombScargle |  | 1.1 (0.9-1.3) | 0.407 | 1.1 (0.9-1.2) | 0.524 |
| Power.Law.Y.Interce pt...Histogram.based |  | 0.9 (0.8-1.1) | 0.444 | 0.9 (0.8-1.1) | 0.547 |
| LF.Power | Low frequency power | 1.2 (0.7-1.8) | 0.523 | 1.1 (0.7-1.7) | 0.562 |
| VLF.Power | Very low frequency power (0.003Hz-0.04Hz) | 1.2 (0.7-1.8) | 0.54 | 1.1 (0.7-1.8) | 0.571 |
| MultiFractal_c1 | Multifractal spectrum cumulant of the first order | 1 (0.9-1.3) | 0.603 | 1.1 (0.9-1.3) | 0.583 |
| MultiFractal_c3 | Multifractal spectrum cumulant of the third order | 0.9 (0.8-1.2) | 0.576 | 0.9 (0.8-1.2) | 0.589 |
| Teo | Teager energy operator average energy | 0.9 (0.8-1.2) | 0.587 | 0.9 (0.8-1.2) | 0.601 |
| LF.HF.ratio.LombSca gle | LF Power lombScargle/HF Power LombScargle | 1 (0.9-1.2) | 0.612 | 1 (0.9-1.2) | 0.614 |
| SymDse_1 | Symbolic dynamics: Shannon enttropy, uniform case | 1 (0.8-1.1) | 0.67 | 1 (0.8-1.1) | 0.617 |
| cDimL |  | 0.9 (0.8-1.2) | 0.583 | 1 (0.8-1.2) | 0.628 |
| histSI |  | 1 (0.8-1.2) | 0.828 | 1 (0.8-1.1) | 0.633 |
| Power.Law.Slope.LombScargle |  | 1 (0.9-1.2) | 0.703 | 1 (0.9-1.2) | 0.647 |
| SymDp1_2 | Symbolic dynamics: percentage of 1 variations sequences, non-uniform case | 1 (0.8-1.2) | 0.754 | 1 (0.8-1.2) | 0.655 |
| Power.Law.X.Interce pt...Frequency.based |  | 1.1 (0.9-1.3) | 0.574 | 1.1 (0.8-1.3) | 0.661 |
| Power.Law.Y.Interce pt.LombScargle |  | 1 (0.9-1.2) | 0.715 | 1 (0.9-1.2) | 0.672 |
| SymDfw_2 | Symbolic dynamics: forbidden words, uniform case | 1 (0.9-1.3) | 0.673 | 1 (0.8-1.3) | 0.71 |
| Skewness | Skewness is a measure of symmetry, or more precisely, the lack of symmetry | 1 (0.8-1.2) | 0.755 | 1 (0.8-1.2) | 0.715 |
| sgridWGT |  | 1 (0.8-1.2) | 0.864 | 1 (0.8-1.2) | 0.731 |
| SymDce_1 | Symbolic dynamics: modified conditional entropy, uniform case | 1 (0.8-1.2) | 0.757 | 1 (0.8-1.2) | 0.734 |
| Sample.entropy | Quantifies the degree of complexity in the input signal. Computed as negative logarithm of estimate of conditional probability that event epochs of length m that match point-wise within tolerance r also match at the next point. The values used are m = 2 and r = 0.15. The r parameter is computed as a percentage of the standard deviation of the event values in the analysis window. | 1 (0.8-1.2) | 0.815 | 1 (0.8-1.2) | 0.76 |
| sedl | Recurrence quantification analysis: Shannon entropy of the diagonals | 1 (0.8-1.2) | 0.813 | 1 (0.8-1.2) | 0.787 |
| fFdP | Fano factor distance from a Poisson distribution | 1 (0.8-1.1) | 0.689 | 1 (0.8-1.2) | 0.789 |
| DM3pb | Dynamical moment of the third order along the principal bisector | 1 (0.9-1.2) | 0.935 | 1 (0.9-1.2) | 0.824 |
| pD | Recurrence quantification analysis: percentage of determinism | 1 (0.8-1.2) | 0.778 | 1 (0.8-1.2) | 0.827 |
| Multiscale.Entropy | Multiscale Entropy | 1 (0.8-1.2) | 0.992 | 1 (0.8-1.2) | 0.834 |
| SymDse_2 | Symbolic dynamics: Shannon entropy, non-uniform case | 1 (0.8-1.2) | 0.913 | 1 (0.8-1.2) | 0.859 |
| dlmax | Recurrence quantification analysis: maximum diagonal line | 1 (0.8-1.2) | 0.932 | 1 (0.8-1.2) | 0.89 |
| Poincaré.SD2 | The Poincaré representaion of an input event series is a scatter plot in which each point is plotted against its neighbour in a Cartesian plane. | 1 (0.9-1.2) | 0.821 | 1 (0.8-1.2) | 0.893 |
| KLPE | Kullback-Leibler permutation entropy | 1 (0.8-1.2) | 0.954 | 1 (0.8-1.2) | 0.893 |
| aFdP | Allan factor distance from a Poisson distribution | 1 (0.8-1.2) | 0.869 | 1 (0.8-1.2) | 0.93 |
| SymDp1_1 | Symbolic dynamics: percentage of 1 variations sequences, uniform case | 1 (0.9-1.2) | 0.787 | 1 (0.8-1.2) | 0.942 |
| fuzEn | Fuzzy entropy | 1 (0.8-1.2) | 0.973 | 1 (0.8-1.2) | 0.946 |
| diffEn | Diffusion entropy | 1 (0.8-1.2) | 0.971 | 1 (0.8-1.2) | 0.982 |
| sevl | Recurrence quantification analysis: Shannon entropy of the vertical lines | 1 (0.8-1.2) | 0.978 | 1 (0.8-1.2) | 0.994 |
| sgridTAU |  | 1 (0.8-1.2) | 0.899 | 1 (0.8-1.2) | 0.997 |
| pL | Recurrence quantification analysis: percentage of laminarity | 1 (0.8-1.2) | 0.983 | 1 (0.8-1.2) | 0.998 |
